# Intrasulcular Restorations of Anterior Teeth According to the BAIR Technique: Evaluation of Periodontal Parameters

**DOI:** 10.3390/dj10030037

**Published:** 2022-03-02

**Authors:** Luca Giachetti, Francesca Cinelli, Michele Nieri

**Affiliations:** Unit of Dentistry, Department of Experimental and Clinical Medicine, University of Florence, Via del Ponte di Mezzo, 48-50127 Firenze, Italy; francescacinelli05@gmail.com (F.C.); michelenieri@gmail.com (M.N.)

**Keywords:** BAIR technique, intrasulcular restorations, composite restorations, direct restorations

## Abstract

Some clinical situations, such as the closure of pronounced diastemas, and the transformation of malformed, small, or peg-shaped teeth, require a rebalancing of dental proportions accompanied by a modification of the gingival contour. A traditional treatment plan can include surgical, prosthetic, and/or orthodontic treatments. In some cases, it can be considered too invasive, and not all patients are willing to undertake long therapies. To overcome these limitations, a possible solution could be the application of the Biologically Active Intrasulcular Restoration (BAIR) technique, which allows us to modify the natural emergence tooth profile using simple intrasulcular direct restorations. The aims of this paper are to investigate possible effects on gingival health, and to assess the patient satisfaction about the aesthetic intervention performed. Periodontal data were collected, and patient satisfaction was registered using the VAS questionnaire. All sites healed without complications, no adverse events were registered or reported by the patients, and no signs of periodontal morbidity were visible. The results show that the patients evaluated the final aesthetics in a positive way, and they perceived a good condition of periodontal health as well. The intervention is felt as almost painless, and patients do not report significant post-operative distress. In conclusion, the BAIR technique can provide a valid therapeutic alternative for patients for whom traditional treatments are not indicated. It is a minimally invasive intervention where both the operating times and the biologic and economic costs are reduced.

## 1. Introduction

The search for aesthetic treatments is common in the routine of dental professionals [1,2]. With the increasing demand for facial aesthetics, patients’ expectations regarding dental treatment have increased. Following this trend, dental patients seek treatment with the primary aim of improving smile aesthetics [3].

The main problems that cause patients to request aesthetic treatments are defects in shape and structure (e.g., peg-shaped teeth, torn teeth, etc.), and closure of the diastemas. The latter is often associated with dento-maxillary disharmonies and microdontism. In situations where there is the need to increase the size of the teeth, and in order to maintain harmonious proportions, it is necessary to lengthen the clinical crown. When it is not possible to lengthen the crown in the incisal direction, it is necessary to find the space in the cervical direction, and then interact with the gingival tissue.

Good management of the gingival parables, as a consequence of the dental modifications, is a key factor for the success of these clinical cases.

The therapeutic response to these needs is mainly conditioned by the age of the patient, as some surgical, implant, or prosthetic interventions, for example, are mainly recommended for adult patients, who have completed skeletal maturation [4]. For this reason, traditional protocols are often not recommended for young patients. In addition to the age limit, these therapeutic protocols require high biological costs, long procedures, as well as high economic costs. They could, in fact, include periodontal interventions to lengthen crowns, orthodontics, and/or prosthodontics. These procedures involve multiple phases, with lengthy clinical visits, and long healing and maturation times of the periodontal tissues [5]. Not all patients are willing to undergo, or can afford, these procedures.

A possible solution to the above-mentioned clinical situations could be the use of the Biologically Active Intrasulcular Restoration (BAIR) [6] technique. It is a low biological cost protocol, which can be performed in a single session. The procedure may also be recommended for young patients. It is non-invasive, it is reversible, and most of the time, it can be performed without anaesthesia. The BAIR technique allows for the correction of aesthetic defects through direct intrasulcular restorations, which permit the modification of the emergence profile of the tooth, and the harmonization of the soft tissues [6].

Considering the advantages of the BAIR technique, especially in terms of invasiveness, it is advisable to investigate the safety of the technique. For years, intrasulcular restorations have sparked debates on possible effects on the health of periodontal tissues [7,8,9,10,11,12]. From this perspective, it is important to evaluate possible complications, as well as to examine the surrounding soft tissues. Scientific evidence suggests that if such intrasulcular restorations are carried out through careful control of all of the clinical steps, and local hygiene is maintained by the patient, then they can integrate perfectly with the surrounding periodontium, without being themselves a cause of inflammation [7,8,9,13,14].

At the same time, it is believed that it is significant to evaluate patient satisfaction too, given that, in the case of aesthetic interventions, this aspect must be a primary objective of the clinician. Patient satisfaction is associated with aesthetic outcomes, psychological traits, and quality of care. In contrast, dissatisfaction levels are associated with treatment time, neuroticism, and poorer pain management [15].

The objective of this two-year follow-up retrospective controlled study was to compare the periodontal variables of BAIR-treated teeth by comparing them with those of untreated control teeth of the same subject. In addition, some Visual Analogue Scale (VAS) variables related to the carried-out intervention were evaluated.

## 2. Materials and Methods

### 2.1. Patient Population

The patient group was examined retrospectively. It consisted of subjects who were consecutively treated with intrasulcular restoration using the BAIR technique at the Department of Experimental and Clinical Medicine, University of Florence, between June 2019 and January 2020. 

Patients included in the study needed aesthetic correction: aesthetic defects in the anterior sector of the maxillary arch, such as peg-shaped or torn teeth; need for alignment of the gingival parables; modification of the dental axes; dento-maxillary disharmony with the need for diastema closure; and transformation of dental elements following agenesis or transposition (e.g., transformation of canine into lateral incisor, or premolar into canine, etc.). All patients gave their written consent to participate in the trial.

Exclusion criteria: patients that at the time of intervention presented Periodontal Screening and Recording (PSR) > 2 (presence of active periodontal disease); patients who suffered from local or systemic diseases that affect the health of periodontal tissues; ongoing therapies with drugs affecting periodontal tissue health; and smoking >10 cig/day.

### 2.2. BAIR Technique

**Figure 1:**
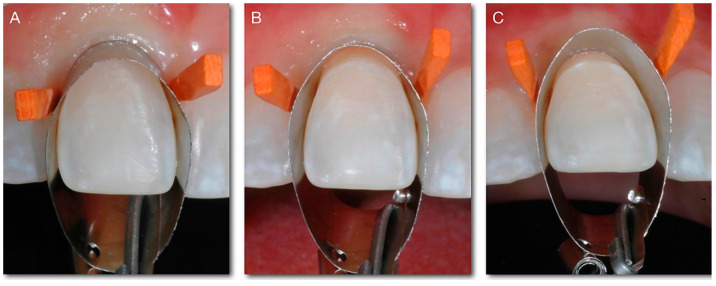
(**A**) The matrix is positioned around the tooth, tilted and pushed in the cervical direction, held adjacent to the tooth, and slid apically to fit the gingival sulcus. (**B**) In this way, it is possible to obtain an isolation of the operative site, and an easy application of the adhesive and composite. (**C**) It is possible to modify the emergence angle, and reconstruct a new “artificial CEJ”.

The proposed method makes use of a simple circular metal matrix, and this allows for the isolation of the operative site, and, at the same time, moves the soft tissues and therefore provides clear access to the intrasulcular portion of the tooth (Figure 1). The layering of the composite allows for the modification of the emergence angle, and the reconstruction of a new “artificial CEJ”. The intrasulcular composite is polymerized in contact with the metal so it is perfectly smooth and cured, since there is no oxygen inhibition of the polymerization. In this way, the intrasulcular part of the restoration does not require finishing and polishing, avoiding surface roughness that can interfere with the adaptation of the periodontal tissues to the restoration. The new emergence profile will guide the soft tissues to adapt in the desired position. The composite is over-layered due to the position and shape of the matrix. The right volumes, in the palate-vestibular direction, are subsequently restored using a subtractive modelling technique [6].

Patients were monitored for about two years, and data collection was made before performing the BAIR technique (T0), immediately after therapy (T1), and at a subsequent follow-up after about two years (T2).

### 2.3. Periodontal Data

PSR and site-specific periodontal variables were collected at baseline: Bleeding on Probing (BOP), Probing Depth (PD), and Plaque Index (PI). BOP and PI: six sites were checked around the tooth, and the result was expressed in x/6. PD is indicated using three values: <3.5 mm, >5.5 mm, or between these two values, for six sites around the tooth. A second site-specific periodontal evaluation was performed at the follow-up (T2). PD is expressed in mm, and PI and BOP are recorded for six sites around the tooth. Each tooth is assigned a value of each variable, corresponding to the worst value recorded among the six sites. The values of the treated tooth are compared with those of the adjacent (control) untreated tooth. In case the central incisor was treated, the lateral is the control; if the lateral is treated, the control is the central. If there are transpositions or agenesis, however, the adjacent tooth is taken as a control. In the event that both central and lateral were treated, only the canine is considered as a control. Periodontal measurements were performed by a calibrated operator (Intraclass Correlation Coefficient (ICC)): 0.87 (95% CI from 0.82 to 0.91).

### 2.4. Patient Satisfaction

The VAS questionnaire (attached in Supplementary Material) was presented before (T0), immediately after the intervention (T1), and about two years later (T2). At T0, it was conducted as an evaluation regarding the perception of aesthetics and gingival health in the anterior sector. At T1, the questionnaire was completed with a question about intraoperative pain and stress related to the intervention. At T2: perception of aesthetics and gingival health regarding treated teeth.

A VAS 1–10 was used to answer the questions, with 1 corresponding to the lower value, and 10 corresponding to the higher value. Both variables in which the highest grade has a positive value (perception of aesthetics and gingival health), and variables in which the highest grade has a negative value (stress, pain), were evaluated. 

The collection of data relating to VAS questionnaires and periodontal charts was carried out by an operator, who was someone other than the one who performed the BAIR intervention. The compilation of the questionnaire by patients was carried out in the absence of operators; however, it was not anonymous.

### 2.5. Variables

The primary variable was PD at two years of follow-up (T2).

The other outcome variables were complications, BoP (T2), and PI (T2). Subjective variables (VAS) were also assessed regarding the treated sector concerning the perception of aesthetics and gingival health.

### 2.6. Statistics

The mean and standard deviations for the quantitative variables, and the frequency and percentages for the qualitative variables, were used as descriptive statistics. The unit of analysis was the tooth.

A statistical model (mixed model) was used for the outcome variable PD at T2 using the patient as a random effect. The group (treatment or control) was the explanatory variable (fixed effect).

Similar models were performed for BoP at T2, and PI at T2.

The difference in VAS between baseline and T2 was assessed with a paired *t*-test. In this case, the unit of analysis was the patient.

The significance threshold is set at 0.05. The statistics were performed with the JMP v. 13.0. 

## 3. Results

**Figure 2:**
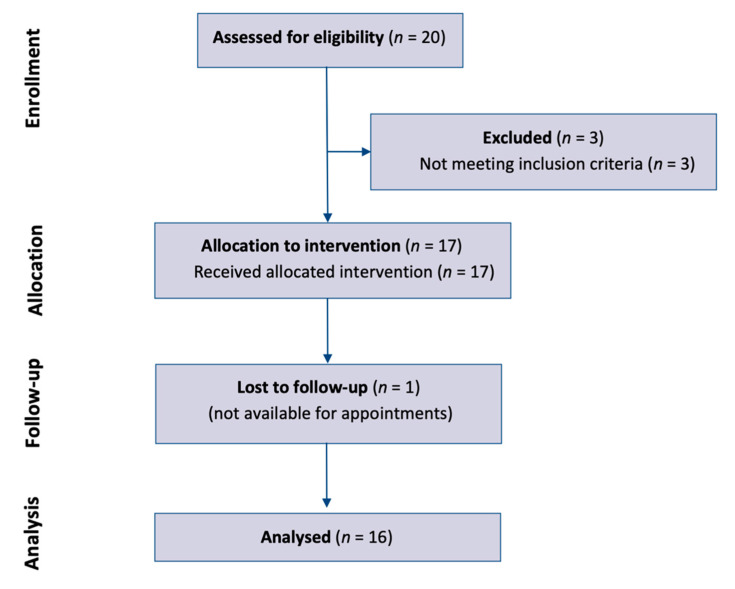
Patient selection process.

Twenty patients had performed BAIR interventions that made them eligible for study (Figure 2). However, from the analysis of the data present in the databases, three of these presented PSR > 2, and were therefore excluded because of exclusion criteria. An additional patient was not included in the study because follow-up data were not available. In conclusion, sixteen patients were included in the study. Age 22.8 ± 14.0 years (from 14.0 to 64.8 years), eleven females (69%) and five males (31%), one smoker (<10 cigarettes) (6%), three with treated diseases (19%), one with familiarity with periodontitis (6%), fourteen who had orthodontics performed in the past (87%), two in orthodontic treatment (12%), and one with abraded incisors (6%). The maximum PSR was 0 in five patients (31%), 1 in four patients (25%), and 2 in seven patients (44%).

Fifty-one teeth (3.2 ± 1.0 teeth per patient) were treated. The treated teeth were all in the upper arch: 21 central incisors (41%), 26 lateral incisors (51%), 2 canines (4%), and 2 first premolars (4%). The control teeth were 32 (2 per patient). The control teeth were also all in the upper arch: 11 central incisors (34%), 19 canines (59%), and 2 first premolars (6%).

### 3.1. Baseline

VAS at baseline and at the end of the treatment are shown in Table 1.

The variables related to the tooth are shown in Table 2. PD is less than 3.5 mm for all sites.

### 3.2. Follow-Up

The follow-up was carried out two years after the execution of the BAIR restorations. All sites healed without complications, and no adverse events were registered or reported by the patients.

The results of the mixed model on periodontal variables are shown in Table 3. The differences are statistically not significant.

The results of the two VAS are shown in Table 4. The differences are significant to the advantage of the measurement at T2.

## 4. Discussion

The BAIR technique allows us to obtain excellent aesthetic results, without having to resort to periodontal, prosthetic, or orthodontic procedures. The results are obtained with a reduced operating time, are immediately visible, and postoperative healing is not required. The restorations performed can be modified at any time, and the intervention is reversible.

**Figure 3:**
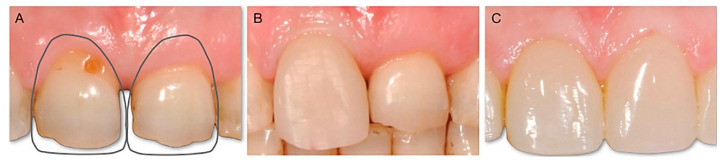
(**A**) A woman in her 50s with worn incisors, and misaligned gingival parables. 1.1 shows root exposure with caries. To solve the aesthetic problem, and “rejuvenate” the smile, it is necessary to enlarge the incisors to close the diastema, align the gingival parables, and find the right proportions. The traditional treatment plan involves surgery to achieve the lengthening of the clinical crown followed by a prosthetic phase. This case was treated in a single session with the BAIR technique. (**B**) Intermediate stage where we can see the 1.1 restored. By changing the emergence profile, and rebuilding a new artificial CEJ more apically, we can obtain the necessary lengthening. (**C**) BAIR restorations finished. The gingival tissues immediately adapted to the new design of the restorations.

**Figure 4:**
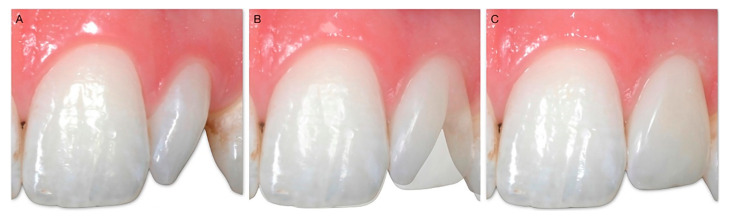
(**A**) A peg-shaped upper left lateral incisor. (**B**) The patient was treated using the BAIR technique. The volume of composite added to restore a shape similar to a normal lateral incisor can be seen in transparency. (**C**) The final result.

**Figure 5:**
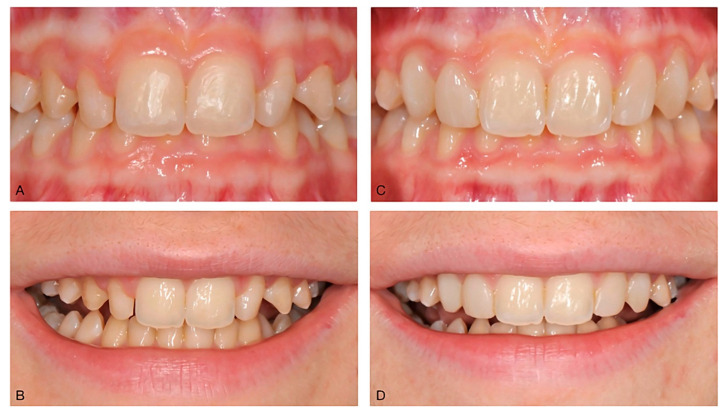
(**A**) 16-year-old girl at the end of orthodontic treatment. There is a noticeable disharmony between the size of the central incisors and those of the lateral incisors and canines (**A**). There are also diastemas that the patient does not like, and which make the smile unpleasant (**B**). In a single session, without the need to give anesthetic, it was possible to enlarge the microdontic teeth, close the diastemas, correct the vestibular inclination of 2.3, and, at the same time, give a better balance to the gingival parables (**C**), thus obtaining a more pleasant smile (**D**).

**Figure 6:**
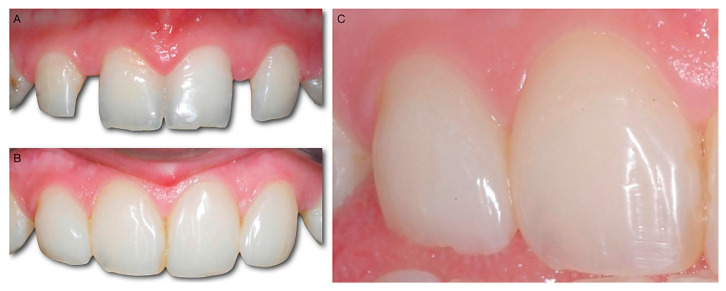
20-year-old man at the end of orthodontic treatment. The frontal sector shows small teeth, and large diastemas (**A**). To meet the aesthetic needs of the patient, it was necessary to enlarge the teeth with intrasulcular restorations, and to recreate the contact points that allowed the papillae to close the unsightly black triangles (**B**). Although the patient does not maintain optimal oral hygiene, the gingival tissues are in excellent health (**C**).

**Figure 7:**
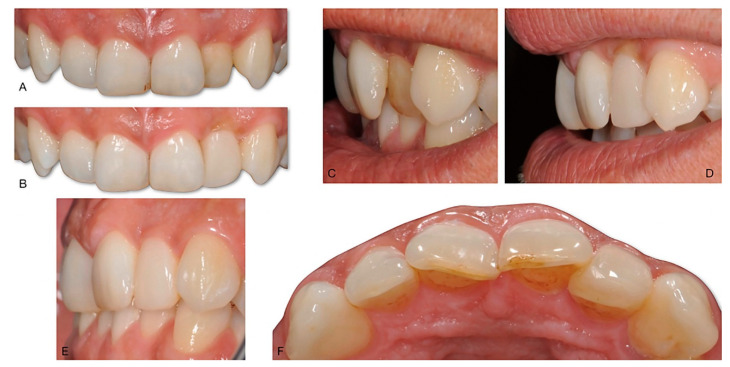
A 37-year-old woman. The 2.2 is tilted towards the palate (**A**,**C**). This tooth, set back compared to the other elements of the front group, is more difficult to manage in home hygiene. Even the self-cleansing mechanisms work worse, and for these reasons, the tooth quickly becomes darker, and creates great discomfort for the patient, who is not willing to undergo orthodontic treatment. The BAIR technique represents an alternative, quick, and possibly reversible solution, with no biological cost, and an immediately visible result (**B**,**D**). It is possible to obtain a remarkable change of the emergence angle in the intrasulcular portion of the restoration, which allows for the reshaping of a lateral incisor congruent with the contralateral one. The follow-up shows a stable result (**E**), and the treated tooth shows an increased thickness (**F**) that does not cause any discomfort for the patient (it should also be considered that the occlusal relationships have not been modified).

The goal of the technique is to change the emergence profile of the tooth by performing intrasulcular restorations. Through the creation of a new “artificial CEJ”, it is possible to modify the angle between the root and the crown both on the vestibular–palatal and on the mesio-distal plane. These changes allow us to guide the positioning of the soft tissues, which are supported by a sort of “shelf” represented by the restoration itself. This allows us not only to change the length of the clinical crown (Figure 3), and correct defects of the shape or structure (Figure 4), but also to harmonize the design of the gingival parables (Figure 5), to close diastema spaces (Figure 6), and to allow for a “virtual” modification of the inclination of the dental axes to improve the smile, and harmonize the gingival profile (Figure 7).

Regarding intrasulcular restorations, there is conflicting evidence in the literature on whether these restorations can cause periodontal damage or inflammation [7,8,9,10,11,12]. The results of this study show that, if the restorations were carried out following the indications, they find a good integration with the soft tissues, without causing gingival inflammation. In the monitored time interval, none of the probed sites exceed the PD > 3.5 mm threshold value. The gum does not show any clinical signs of inflammation, nor any kind of “discomfort” is reported by the patients themselves. The periodontal values recorded at the end of the observation period are comparable to those of the adjacent untreated tooth.

These claims are confirmed in numerous studies [7,8,9,13,14] that report the possibility of making subgingival restorations, without this being considered the cause of periodontal inflammation. Some authors [16,17], on the basis of histological examinations, report the possibility of the formation of an epithelial attachment on different materials: 4-META/MMA-TBB resin [4-(2-methacryloxy-ethyl) trimellitic anhydride/methyl methacrylate-tributylborane], and resin-ionomer restorative materials. Frese [7] proposes the possibility that there may be an epithelial attachment between the resin composite and the gingiva. Dragoo [17] states that there may be the possibility of formation of both epithelial attachment and a connective tissue adhesion between deep periodontium and resin ionomer restoration materials. This evidence supports the fact that the materials used are perfectly biocompatible [11,16,18], and that, therefore, they can also be used within the gingival sulcus, without this being the cause of periodontal damage.

As stated above, there are many factors that contribute to the long-term success of intrasulcular restoration. Since the composite during layering and subsequent polymerization comes into contact with the matrix itself, this excludes contact with oxygen which could otherwise prevent its adequate polymerization [6,19]. The matrix is used as a “mold” for the apical portion of the restoration; it will not require subsequent finishing, which could induce the formation of superficial roughness that could interfere with the periodontal health [20,21]. The matrix correctly positioned in the sulcus also controls humidity, and allows us to eliminate the necessity of the dental dam. [6] The dental dam would not, however, allow for the isolation of the most apical margin of the restoration: neither an adequate view nor management of gingival levels during the intervention. However, the use of devices that allow for the dislocation of the lips and cheeks is recommended for better control of the operating field. It is understood that maintaining excellent oral hygiene remains important for good results even in the long term [7,13].

In addition to periodontal health, the aim of the study was to analyze patient satisfaction regarding the intervention performed. It must be considered that the patients included in the study were treated for aesthetic needs, and the maxillary anterior teeth are a key aesthetic component of a smile [22]. Obtaining a good level of satisfaction contributes to the success of the treatment plan. The results show that the patients evaluated the final aesthetics in a positive way (9.1 ± 1.0), and there is also a marked improvement compared to the initial aesthetics evaluation (4.1). Patients perceived a good state of periodontal health as well (8.4 ± 1.0). The intervention was felt as almost painless (3.6 ± 1.9), and patients did not report important post-operative distress (2.8 ± 1.5).

The positive scores are probably related to the fact that the BAIR technique is considered non-invasive by the patient. Compared to the therapeutic alternatives, this protocol does not require recourse to surgery, and as such, healing and settlement times are not necessary. In most cases, it is not necessary to give anaesthetic. In addition, the results are achieved in a single appointment.

The VAS recordings allow us to focus on the fact that daily dentistry should care about the values, expectations, and preferences of patients. In recent decades, the Evidence-Based Dentistry model has increasingly established itself. Standardization, however, often does not consider the important differences in situations that distinguish one patient from another. The therapy should be rather individualized, allowing the therapy to become the most appropriate for the patient and for their specific situation [23,24,25]. The overall value of the intervention, coordinated with patient satisfaction, can be evaluated as the quotient between “quality” (outcomes, services, safety) and “costs” (financial, but also biological, time, psychological, opportunity costs). The BAIR technique allows for the reduction of all of the “costs” related to the intervention, increasing the treatment’s overall value. It must also be kept in mind that every patient has different expectations and aspirations regarding quality and costs. This leads us to consider “Value-Based Dentistry” as an extension of Evidence-Based care to meet patient-centered objectives [26].

Another aspect of the BAIR technique that makes it attractive for the patient is the possibility of being able to return to the initial state. This is certainly not possible with traditional therapies that do not allow us to manage situations in which the patient is not fully satisfied. In the case of the BAIR technique, you can change the result, or return to the initial situation, simply by removing the added composite.

Some limitations have to be considered due to the nature of the study: the results are presented in the mid-term via an observational study. The future target is to continue monitoring the patients to obtain long-term results, in particular with randomized trials.

## 5. Conclusions

The BAIR technique can provide a valid therapeutic alternative for patients for whom traditional treatments are not recommended, or which are so demanding that patients are unwilling to accept them.

In fact, it is a minimally invasive intervention where both the operating times and the biologic and economic costs are reduced. 

The data relating to this preliminary study show a good integration of the restorations with the surrounding periodontium. Periodontal data should also be thoroughly investigated in the long-term through randomized trials. Results from the VAS questionnaires show an excellent acceptance by the patients of the performed intervention in terms of the perception of aesthetics and gingival health. The discomfort associated with the therapy (pain, stress) is minimal.

## Figures and Tables

**Table 1:** VAS at baseline and at treatment.

VARIABLE	*N* = 16
VAS Aesthetics T0	5.0 ± 2.2
VAS Gingival Health T0	6.7 ± 1.8
VAS Intra-op Pain T1	3.6 ± 1.9
VAS Intra-op Stress T1	2.8 ± 1.5

**Table 2:** Variables related to the tooth at baseline.

VARIABLE	*N* = 51
BoP (sites)	0.4 ± 1.0
PI (sites)	0.3 ± 0.7

**Table 3:** Results at follow-up (T2).

VARIABLE	Treated Group *N* = 51	Control Group *N* = 32	Difference	95%CI	*p*-Value *
PD at follow-up (mm)	2.4 ± 0.6	2.4 ± 0.6	0.1	−0.2; 0.3	0.6777
BOP (sites)	0.4 ± 0.8	0.5 ± 1.0	−0.1	−0.5; 0.3	0.6215
PI (sites)	1.7 ± 1.3	1.4 ± 1.5	0.3	−0.4; 0.9	0.4089

* Mixed models.

**Table 4:** VAS results.

VARIABLE	T2 *N* = 16	T2-T0 *N* = 16	95%CI	*p*-Value *
VAS Aesthetics	9.1 ± 1.0	4.1	2.9; 5.3	<0.0001
VAS Gingival Health	8.4 ± 1.0	1.7	0.6; 2.8	0.0045

* Paired *t*-test.

## Data Availability

The data presented in this study are available on request from the corresponding author.

## Supplementary Materials

The following supporting information can be downloaded at: https://www.mdpi.com/article/10.3390/dj10030037/s1, VAS Questionnaire.
