# Supplementary material for: Intrasulcular Restorations of Anterior Teeth According to the BAIR Technique: Evaluation of Periodontal Parameters
Source: Dent J (Basel). 2022 Mar 2;10(3):37. doi: 10.3390/dj10030037 (PMC8947544; doi:10.3390/dj10030037)
Supplement: Supplementary file 1 [file dentistry-10-00037-s001.zip › dentistry-1512083-supplementary.pdf]

VAS Questionnaire

- **T0**

VAS Aesthetics
Come valuti l'estetica dei denti anteriori? Dai un voto da 1 a 10, dove 1 significa "pessima estetica" e 10 significa "estetica eccellente".
How do you evaluate the aesthetics of the anterior teeth? Rate it from 1 to 10, where 1 means "bad aesthetics" and 10 means "excellent aesthetics".

1	2	3	4	5	6	7	8	9	10

VAS Gingival Health
Come valuti la salute delle gengive intorno ai denti anteriori? (Sangue, dolore, infiammazione) Dai un voto da 1 a 10, dove 1 significa "pessima salute" e 10 significa "ottima salute".
How do you assess the health of the gums around the anterior front teeth? (Blood, pain, inflammation) Rate it from 1 to 10, where 1 means "very poor health" and 10 means "excellent health".

1	2	3	4	5	6	7	8	9	10

- **T1**

VAS Intra-op Pain
L'intervento è stato doloroso? Dai un voto da 1 a 10, dove 1 significa "nessun dolore" e 10 significa "dolore insopportabile".
Was the surgery painful? Rate it from 1 to 10, where 1 means "no pain" and 10 means "unbearable pain".

1	2	3	4	5	6	7	8	9	10

VAS Intra-op Stress T1
L'intervento è stato stressante? Dai un voto da 1 a 10, dove 1 significa "nessuno stress" e 10 significa "stress insopportabile".
Was the surgery stressful? Rate it from 1 to 10, where 1 means "no stress" and 10 means "unbearable stress".

1	2	3	4	5	6	7	8	9	10

- **T2**

VAS Aesthetics
Come valuti l'estetica dei denti anteriori dopo l'esecuzione delle ricostruzioni? Dai un voto da 1 a 10, dove 1 significa "pessima estetica" e 10 significa "estetica eccellente".
How do you evaluate the aesthetics of the anterior teeth after the reconstructions have been performed? Rate it from 1 to 10, where 1 means "bad aesthetics" and 10 means "excellent aesthetics".

1	2	3	4	5	6	7	8	9	10

VAS Gingival Health

Come valuti la salute delle gengive intorno ai denti anteriori trattati? (Sangue, dolore, infiammazione) Dai un voto da 1 a 10, dove 1 significa ″pessima salute″ e 10 significa ″ottima salute″.

How do you assess the health of the gums around the treated front teeth? (Blood, pain, inflammation) Rate it from 1 to 10, where 1 means "very poor health" and 10 means "excellent health".

1	2	3	4	5	6	7	8	9	10
